# Contribution of TRPC3 to store-operated calcium entry and inflammatory transductions in primary nociceptors

**DOI:** 10.1186/1744-8069-10-43

**Published:** 2014-06-26

**Authors:** Hazim Alkhani, Ariel R Ase, Rebecca Grant, Dajan O’Donnell, Klaus Groschner, Philippe Séguéla

**Affiliations:** 1 Department of Neurology and Neurosurgery, Montreal Neurological Institute and Alan Edwards Research Centre for Pain, McGill University, Montreal, QC, Canada; 2 AstraZeneca R&D Montreal, Ville Saint-Laurent, QC, Canada; 3 Institute of Biophysics, Medical University of Graz, Graz, Austria; 4 Montreal Neurological Institute, 3801 University, Suite 778, H3A 2B4 Montreal, QC, Canada

**Keywords:** TRPC channel, Nucleotide, ATP, Protease, GPCR, Phospholipase C, Sensory neuron, Sensitization, DRG, Trigeminal, Inflammation, Pain

## Abstract

**Background:**

Prolonged intracellular calcium elevation contributes to sensitization of nociceptors and chronic pain in inflammatory conditions. The underlying molecular mechanisms remain unknown but store-operated calcium entry (SOCE) components participate in calcium homeostasis, potentially playing a significant role in chronic pain pathologies. Most G protein-coupled receptors activated by inflammatory mediators trigger calcium-dependent signaling pathways and stimulate SOCE in primary afferents. The aim of the present study was to investigate the role of TRPC3, a calcium-permeable non-selective cation channel coupled to phospholipase C and highly expressed in DRG, as a link between activation of pro-inflammatory metabotropic receptors and SOCE in nociceptive pathways.

**Results:**

Using *in situ* hybridization, we determined that TRPC3 and TRPC1 constitute the major TRPC subunits expressed in adult rat DRG. TRPC3 was found localized exclusively in small and medium diameter sensory neurons. Heterologous overexpression of TRPC3 channel subunits in cultured primary DRG neurons evoked a significant increase of Gd^3+^-sensitive SOCE following thapsigargin-induced calcium store depletion. Conversely, using the same calcium add-back protocol, knockdown of endogenous TRPC3 with shRNA-mediated interference or pharmacological inhibition with the selective TRPC3 antagonist Pyr10 induced a substantial decrease of SOCE, indicating a significant role of TRPC3 in SOCE in DRG nociceptors. Activation of P2Y2 purinoceptors or PAR2 protease receptors triggered a strong increase in intracellular calcium in conditions of TRPC3 overexpression. Additionally, knockdown of native TRPC3 or its selective pharmacological blockade suppressed UTP- or PAR2 agonist-evoked calcium responses as well as sensitization of DRG neurons. These data show a robust link between activation of pro-inflammatory receptors and calcium homeostasis through TRPC3-containing channels operating both in receptor- and store-operated mode.

**Conclusions:**

Our findings highlight a major contribution of TRPC3 to neuronal calcium homeostasis in somatosensory pathways based on the unique ability of these cation channels to engage in both SOCE and receptor-operated calcium influx. This is the first evidence for TRPC3 as a SOCE component in DRG neurons. The flexible role of TRPC3 in calcium signaling as well as its functional coupling to pro-inflammatory metabotropic receptors involved in peripheral sensitization makes it a potential target for therapeutic strategies in chronic pain conditions.

## Background

The transient receptor potential (TRP) gene superfamily consists of a large set of tetrameric channels permeable to monovalent and/or divalent cations. In mammals, several of the 28 TRP channel family members are expressed in subpopulations of peripheral sensory neurons and are involved in the transduction of thermal, mechanical and chemical stimuli, with documented roles in normal and pathological nociception [1].

However, both the cellular physiology of TRP canonical (TRPC) channels in sensory neurons and their exact role in nociception have yet to be clarified. The TRPC subtypes are non-selective calcium-permeable cationic channels ubiquitously expressed in most tissues. They integrate several types of intracellular stimuli, including PLC and PKC activity, DAG levels, intracellular calcium levels and PIP_2_ levels into changes in membrane potential and calcium entry [2]. Of particular interest is the role of TRPC3 in somatosensory pathways because of recent evidence pointing to an almost exclusive expression of this channel in IB4+ nociceptors, a large population of C-fiber nonpeptidergic sensory neurons known to contribute to inflammatory pain [3].

One of the hallmarks of inflammatory pain is the sensitization of peripheral nociceptors innervating the inflamed area. During the inflammation process, induced either by chemical agents, injury or infection, a stimulus-response shift is observed in nociceptors, resulting in an enhanced response to noxious stimuli (hyperalgesia) or in a nocifensive response to normally innocuous stimuli (allodynia) [4]. The many chemicals released at the site of inflammation form a “soup” that triggers oedema, itchiness, redness, and sensitization [5]. This “inflammatory soup” consists of a diverse set of signaling molecules which are capable of inducing hyperexcitability by activating their cognate membrane receptors at the surface of nociceptors [6]. Many of these receptors include well characterized Gq-protein coupled receptors (GqPCR), such as the P2Y2 purinoceptor, bradykinin B2 and protease-activated PAR2 receptor [6,7]. The coupling of these GqPCRs to the phospholipase C (PLC) pathway produces two bioactive compounds upon stimulation, diacylglycerol (DAG) and inositol trisphosphate (IP_3_), both of which act to increase intracellular calcium levels through two distinct mechanisms: one is referred to as store-operated calcium entry (SOCE) and involves the activation of calcium channels by IP_3_-induced depletion of endoplasmic reticulum calcium stores. The other, receptor-operated calcium entry (ROCE), involves the activation of calcium-permeable channels directly by DAG [8-11]. Hence, the production of these two secondary messengers via PLC activation results in both intracellular calcium levels increase and enhanced entry of extracellular calcium across the plasma membrane following depletion of calcium stores. Crucially, one common resulting action of these two pathways is the downstream activation of calcium-dependent protein kinase C (PKC) isoforms mediating the phosphorylation and sensitization of voltage- and ligand-gated channels involved in acute and chronic inflammatory responses [12,13].

The signaling pathways of known pro-inflammatory receptors in DRG neurons such as P2Y2 and PAR2 are not completely characterized, but an increase in intracellular calcium levels is critical for the activation of their downstream effectors [8,10,14]. It has recently been shown that an abnormal persistent increase in intracellular calcium levels mediates the transition from acute to chronic pain in inflammatory pancreatitis [15,16]. Thus the regulation of intracellular calcium levels, primarily through SOCE, could be a key mechanism in preventing sensitization. While most TRPC channel subtypes play a role in ROCE, some members of the TRPC family have been implicated in SOCE signaling as well [17,18]. Moreover, members of the TRPC3/6/7 channel subfamily have been shown to be functionally coupled to the PLC pathway in various mammalian cell lines [19-22]. In addition, TRPC channels have been linked to the activation of pro-inflammatory bradykinin B2 receptors in non-neural cells [23,24], suggesting that this class of calcium-permeable cation channels might be involved in both SOCE and ROCE signaling mechanisms downstream of the PLC pathway in neurons as well.

The present report focuses on the ill-defined role of TRPC channels in mammalian sensory pathways. Our data show that TRPC3, highly expressed in adult rodent DRG, is coupled to several classes of pro-inflammatory metabotropic receptors and plays a significant role in calcium homeostasis and sensitization in primary nociceptors, through its involvement in both SOCE and ROCE.

## Results and discussion

### Expression of the TRPC gene family in rat DRG

**Figure 1:**
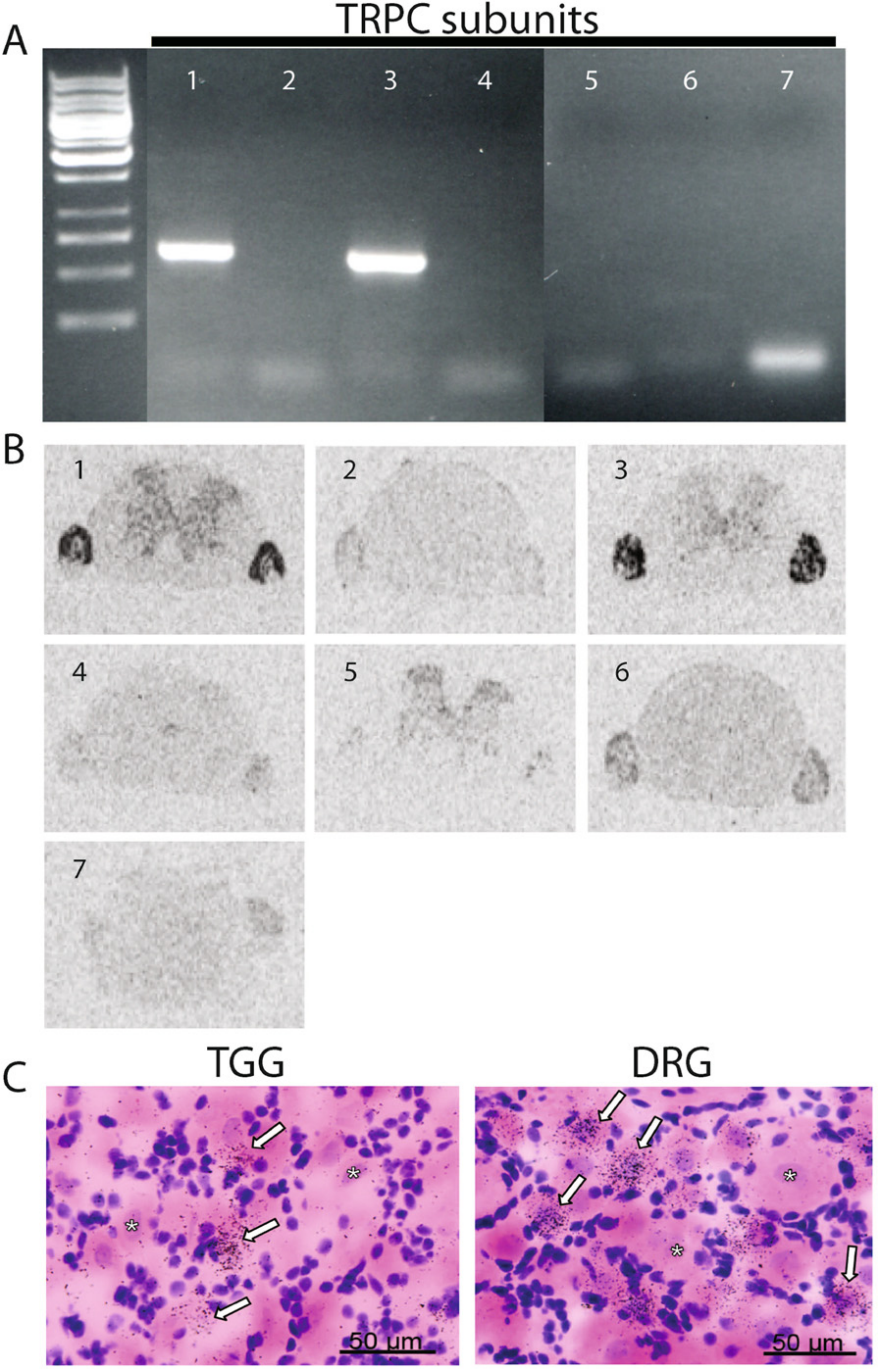
**TRPC family gene expression in sensory ganglia. (A)** TRPC mRNA detection using a RT-PCR screen against TRPC1-7 in adult rat DRG. TRPC1 and TRPC3 are the major subunits expressed in DRG, along with low levels of TRPC6 and little or no signal for TRPC 2, 4, 5 and 7. **(B) ***In situ* expression profile of all TRPC family members at the whole DRG and spinal cord level. TRPC1 and TRPC3 are expressed at high levels in DRGs, with minimal detection in the spinal cord. Low but significant TRPC6 expression is also observed at the DRG level. **(C)** Emulsion staining with cellular resolution clearly shows that TRPC3 mRNA is localized in a subpopulation of small and medium diameter neurons (arrows) in DRG and trigeminal ganglia (TGG). Representative negative small-diameter and large-diameter neurons are also indicated (stars). Scale bar = 50 μm.

To determine the expression profile of TRPC subunits in adult rat DRG, RT-PCR was performed in order to potentially detect all 7 mammalian TRPC members (TRPC1-7). As shown in Figure 1A, TRPC1 and TRPC3 genes appear to be strongly transcribed, while other TRPC subunit mRNAs were found at low levels or not at all. To determine the level of TRPC expression using a complementary approach, we performed *in situ* hybridization targeting each of the TRPC transcripts in rat spinal sections containing the DRGs. As illustrated in Figure 1B, we confirm that TRPC1 and TRPC3 are the only two major transcripts in the TRPC family present at high levels in DRGs. Furthermore, mRNA detection *in situ* with cellular resolution in DRG and trigeminal ganglia sections allowed us to assess that TRPC3 expression is mainly confined to the subpopulation of small and medium diameter sensory neurons (Figure 1C), the vast majority of which are C-fiber nociceptors, in agreement with findings in the mouse somatosensory pathways published recently [18,25,26]. TRPC1 function has been linked to ubiquitous STIM1-dependent SOCE [27-29] and our histological data indicate that its expression is not restricted to a specific population of sensory neurons in DRG, therefore we focused our investigation on DAG-gated TRPC3 channels localized in primary nociceptors.

### TRPC channels are involved in SOCE in DRG neurons

**Figure 2:**
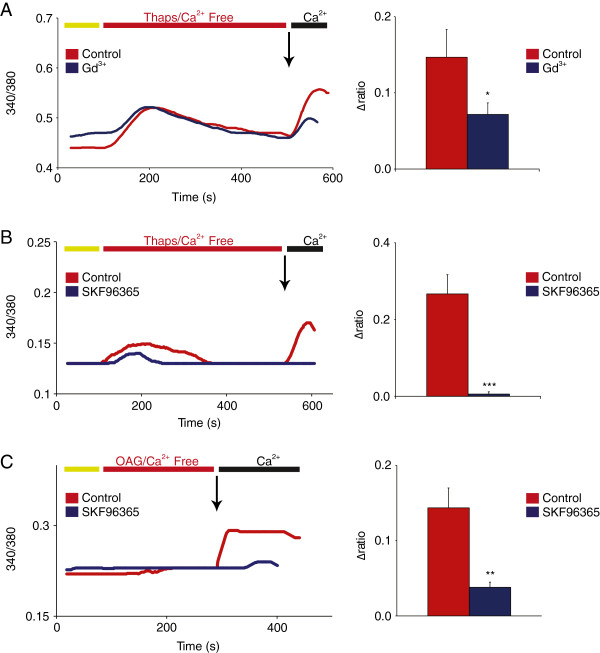
**Contribution of TRPC channels to SOCE in DRG neurons.** All traces are representative calcium responses recorded using the Fura-2 ratiometric method. **(A)** Thapsigargin (Thaps, 1 μM, 7 min) evokes a biphasic SOCE response in cultured rat DRG neurons (control). The first phase reflects the release of calcium from ER stores while the second phase reflects the influx of extracellular calcium, upon calcium re-addition to the perfusion solution (calcium add-back protocol), via Orai and/or TRPC channels. The addition of the Orai blocker Gd^3+^ (1 μM, 7 min) resulted in a significant decrease of calcium influx by 50%, suggesting a possible role for other channels including TRPCs in the remaining response (n = 8-19, P < 0.05). **(B)** SKF96365 (30 μM), a general SOCE and TRPC blocker, completely blocks SOCE (n = 14-23, P < 0.0005). **(C)** Effect of SKF96365 on TRPC3 activity. Activation of TRPC3 with the DAG analog OAG (50 μM, 3 min), is significantly inhibited by SKF96365 (approximately 75%), indicating a major role for TRPC3 in the TRPC component of the thapsigargin-induced SOCE response observed in DRG neurons (n = 15-18, P < 0.01).

The activation of the PLC pathway by pro-inflammatory GPCRs leads to the production of cytosolic IP_3_ that, through the gating of its receptor-channel IP_3_R located at the surface of the ER, induces the release of calcium from intracellular stores. This initiates SOCE, the primary mechanism responsible for calcium homeostasis, through the activation of the ER transmembrane calcium sensors STIM1 and STIM2. Activated STIM proteins aggregate with each other and translocate to the plasma membrane where they interact with several types of calcium channels, including the ubiquitous Orai1 calcium-selective channel and specific TRPC channel subtypes, inducing them to open their gates and causing an influx of extracellular calcium, further increasing intracellular calcium levels and replenishing ER calcium stores. This increase in intracellular calcium levels is thought to contribute to the activation of calcium-sensitive switches and pathways that mediate inflammatory hypersensitisation, most notably PKC [30,31]. To determine if TRPC channels play a role in this response, we first assessed the presence of SOCE in cultured DRG neurons by treating them with 1 μM thapsigargin for 7 minutes in calcium-free solution, leading to passive diffusion of ER calcium into the cytoplasm (Figure 2A), and hence mimicking the physiological process of store-depletion and the activation of SOCE. The addition of the Orai blocker Gd^3+^ at 1 μM to the perfusion solution clearly produced a robust decrease in calcium influx of approximately 50% (Δ340/380 = 0.147 ± 0.034 for control vs. 0.072 ± 0.015 for Gd^3+^ condition), as shown in Figure 2A. The remaining calcium influx may indicate the involvement of TRPC channels. To test if TRPC channels contribute to SOCE in DRGs, we used the generic TRPC and SOCE pharmacological blocker SKF96365 at 30 μM for 50 minutes. The addition of SKF96365 completely abolishes SOCE (Δ340/380 = 0.267 ± 0.050 for control vs. 0.017 ± 0.006 for SKF96365), providing evidence for the recruitment of TRPC channels in the SOCE response in rat DRGs (Figure 2B). Additionally, the activation of TRPC3 by the DAG analog OAG (50 μM, 3 min) is also potently inhibited by SKF96365 (Δ340/380 = 0.144 ± 0.026 for control vs. 0.038 ± 0.007 for SKF96365, Figure 2C), suggesting a specific role for DAG-gated TRPC3 channels in the SOCE response observed.

### Contribution of TRPC3 to SOCE in DRG neurons

**Figure 3:**
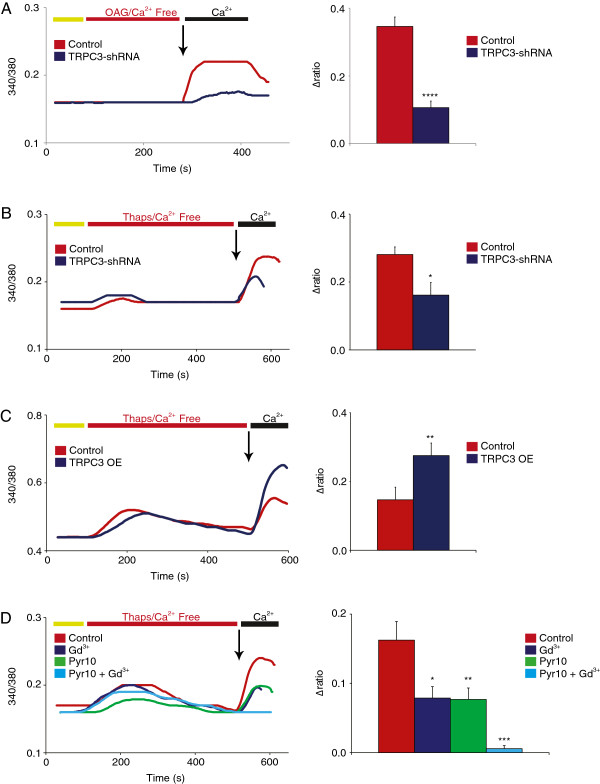
**TRPC3 participates in SOCE in DRG neurons. (A)** TRPC3 shRNA-mediated knockdown was validated in a functional assay to determine the inhibitory effect of the shRNA on OAG-evoked calcium entry in DRG neurons (n = 9-11, P < 0.0001). **(B)** shRNA-mediated knockdown of TRPC3 in the SOCE assay clearly shows a strong contribution of this channel to the overall calcium influx. A decrease of approximately 42% is observed (n = 13-27, P < 0.05). **(C)** Heterologous overexpression of TRPC3 (OE) in DRG neurons produced a drastic increase in SOCE-mediated calcium influx of approximately 85% (n = 16-19, P < 0.01). **(D)** The inhibition of TRPC3 activity by the selective blocker Pyr10 (10 μM, 15 min) also resulted in a strong decrease of SOCE activity, with calcium influx dropping approximately 50% (n = 34-39, P < 0.01). The cumulative inhibition of both Orai and TRPC3 with Gd^3+^ and Pyr10, respectively, almost completely abolished SOCE response in DRG (n = 18-21, P < 0.001).

TRPC3 has been implicated in SOCE in pancreatic cells [32], prostate smooth muscle cells [33], HEK-293 cells [34], and hippocampal neuronal cells [35]. To check if TRPC3 has any role in SOCE response in DRG neurons, we opted for RNA interference with a subunit-specific GFP-tagged TRPC3 shRNA. Its knockdown efficacy was measured at close to 70% in OAG-evoked TRPC3-mediated calcium responses in heterologous expression (Δ340/380 = 0.346 ± 0.028 for control vs. 0.106 ± 0.019 for TRPC3 shRNA-tranfected cells, Figure 3A). Once validated, this TRPC3 shRNA was transfected into cultured primary DRG neurons that were recorded 4-5 days post transfection. With respect to controls, TRPC3 transcript knockdown reduced SOCE response by approximately 42% (Δ340/380 = 0.281 ± 0.022 for control vs. 0.161 ± 0.037 for TRPC3 shRNA, Figure 3B). Conversely, overexpressing TRPC3 (TRPC3 OE) by transfecting DRG neurons with TRPC3 cDNA produced a large increase in store-mediated calcium influx (Δ340/380 = 0.147 ± 0.034 for control vs. 0.275 ± 0.037 for TRPC3 OE, Figure 3C). Application of the TRPC3 blocker Pyr10 (10 μM, 15 min) inhibited store-dependent calcium influx by approximately 50% (Δ340/380 = 0.162 ± 0.027 for control vs 0.077 ± 0.016 for Pyr10, Figure 3D), mirroring our shRNA knockdown results. Furthermore, the addition of both the Orai blocker Gd^3+^ and TRPC3 blocker Pyr10 almost completely abolished SOCE (Δ340/380 = 0.162 ± 0.027 for control vs. 0.006 ± 0.004 for Pyr10 + Gd^3+^), indicating that the two major components of SOCE in DRG nociceptors are the Orai and TRPC3 channels (Figure 3D). This is the first direct evidence showing the major role that TRPC3 plays in SOCE response in DRG sensory neurons. However, the role of TRPC3 as a SOCE channel has been controversial [32,35-38]. As TRPC channels integrate many intracellular signals, these discrepancies could simply be related to the intracellular environment of different cell types and/or to species specificities. We report here a strong contribution of TRPC3 to SOCE in rat DRG neurons by showing store-calcium fluxing, in addition to its presumed role in ROCE. This bi-modal functionality of TRPC3 makes it a versatile and unique channel subtype involved in calcium homeostasis and calcium-dependent signaling.

### TRPC3 involvement in P2Y2 receptor transduction

**Figure 4:**
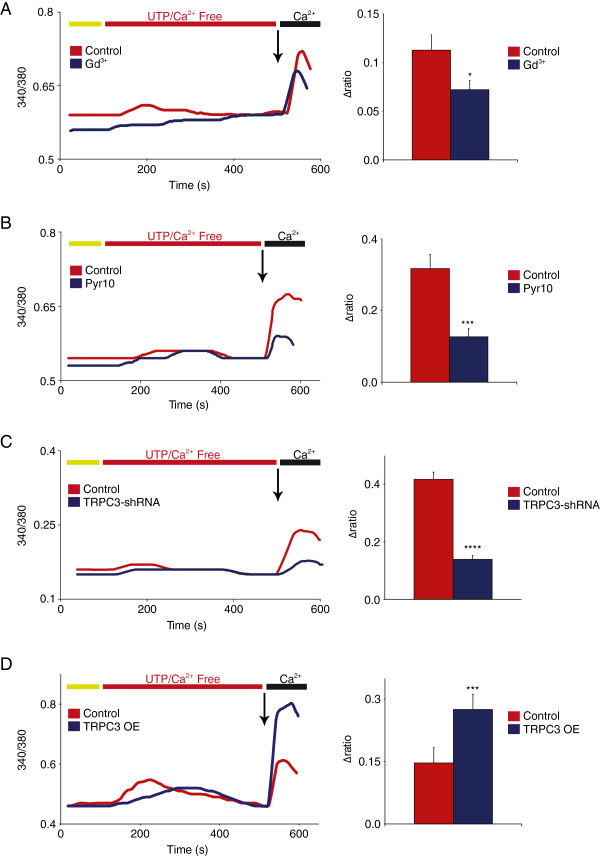
**Functional coupling between TRPC3 and UTP/P2Y2 signaling in DRG neurons. (A)** The addition of 100 μM UTP for 7 minutes to the calcium-free perfusion solution activates P2Y2 receptors, which initiate both SOCE and ROCE responses. The addition of Gd^3+^ removes the Orai component of the UTP-evoked response, which accounts for approximately 35% of the overall calcium influx (n = 17-19, P < 0.05). **(B)** The blockade of TRPC3 specifically with Pyr10 (10 μM, 15 min) resulted in a drastic decrease of UTP-evoked calcium entry, resulting in 60% inhibition (n = 25-28, P < 0.001). **(C)** shRNA-mediated knockdown of TRPC3 induced in similar decrease of P2Y2-mediated calcium entry (n = 34-46, P < 0.0001). **(D)** Heterologous overexpression of TRPC3 (OE) resulted in 90% increase of calcium influx in the UTP response, indicating a strong link between TRPC3 activity and P2Y2 transduction (n = 24-45, P < 0.001).

After determining that TRPC3 contributes to SOCE, we sought to investigate its link to pro-nociceptive pathways. The activation of the purinoceptor P2Y2 by pro-inflammatory nucleotides such as ATP or UTP initiates the PLC pathway through Gq-coupling, activating both SOCE and ROCE by generating IP_3_ and DAG, respectively. To examine if TRPC3 contributes to P2Y2-mediated calcium signaling, we applied 100 μM UTP, a selective agonist for P2Y2 receptors [39], on primary DRG cultures for 7 minutes. The addition of Gd^3+^, and hence blockade of Orai function, produced a decrease of calcium influx upon re-addition of calcium to the perfusion solution. However, in these conditions, Orai contribution appears to be limited at about 35% of the overall calcium entry evoked by UTP (Δ340/380 = 0.113 ± 0.016 for control vs. 0.072 ± 0.009 for Gd^3+^, Figure 4A). Application of the TRPC3 antagonist Pyr10 decreased P2Y2-mediated calcium entry by as much as 60% (Δ340/380 = 0.317 ± 0.039 for control vs. 0.127 ± 0.023 for Pyr10, Figure 4B). This significant contribution of TRPC3 to UTP-evoked calcium responses in DRG neurons was confirmed. Using a TRPC3 shRNA construct for knockdown, where a similar decrease in calcium entry was observed (Δ340/380 = 0.417 ± 0.025 for control vs. 0.139 ± 0.013 for TRPC3 shRNA-treated cells, Figure 4C). Confirming a functional link between TRPC3 and P2Y2 receptor activity, overexpression of TRPC3 subunits in DRG neurons induced a significant increase in UTP-evoked calcium influx (Δ340/380 = 0.135 ± 0.026 for control vs. 0.257 ± 0.025 for TRPC3 OE), as shown in Figure 4D. These results clearly indicate that TRPC3 plays a major role in P2Y2 function, and they substantiate for the first time a robust functional link between TRPC3 and the pro-inflammatory nucleotides ATP/UTP in peripheral pain pathways.

### Significant contribution of TRPC3 to PAR2-mediated calcium signaling

**Figure 5:**
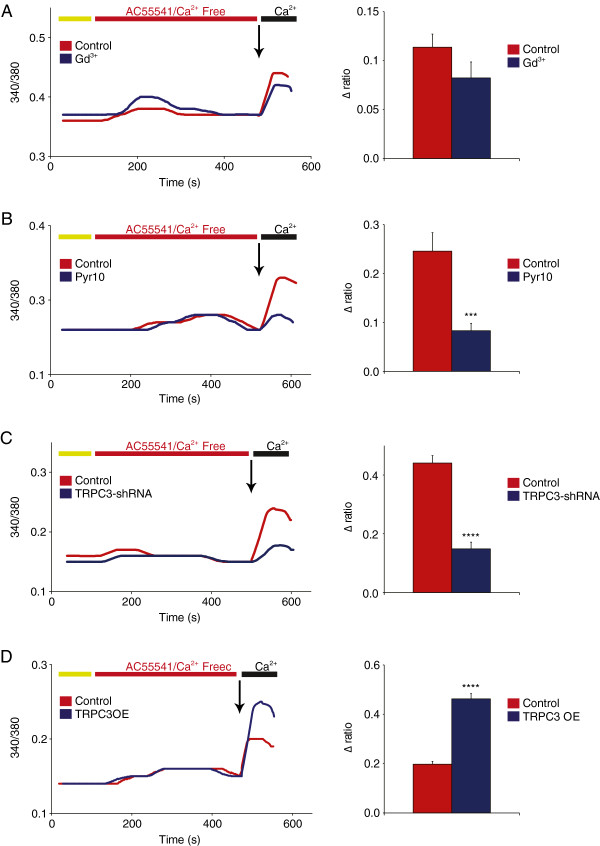
**TRPC3 is linked to proteases/PAR2 transduction in DRG neurons. (A)** Addition of 100 μM AC55541 to the calcium-free perfusion solution activates PAR2 receptors, which initiate both SOCE and ROCE responses. Treatment with Gd^3+^ removed the Orai component, which produced a small but non-significant decrease in calcium influx (n = 22-24, P > 0.05). **(B)** The selective inhibition of TRPC3 with Pyr10 (10 μM, 15 min) resulted in a drastic decrease (66%) of AC55541-evoked calcium entry (n = 19-29, P < 0.001). **(C)** shRNA-mediated knockdown of TRPC3 induced a similar decrease in PAR2-mediated calcium entry of approximately 66% (n = 24-73, P < 0.0001). **(D)** Heterologous overexpression of TRPC3 (OE) increased by 135% the calcium influx following PAR2 activation, indicating a strong link between TRPC3 activity and PAR2 function (n = 18-26, P < 0.0001).

To further generalize the notion of an association of TRPC3 with pro-inflammatory mediators, we sought to test a functional link of TRPC3 to another metabotropic receptor involved in peripheral sensitization in inflammatory conditions, the Gq-coupled protease-activated receptor 2 (PAR2). Much like P2Y2, PAR2 is also coupled to the PLC pathway and to a rise in intracellular calcium levels leading to the activation of PKC and other calcium sensitive processes, which are thought to contribute significantly to inflammatory sensitization [40-42]. We stimulated cultured DRG neurons with the PAR2 selective agonist AC55541 at 100 μM for 7 minutes along with the Orai blocker Gd^3+^. Contrary to the results with P2Y2, the Orai calcium channels did not appear to be a major source of calcium influx in the PAR2-mediated response (Δ340/380 = 0.114 ± 0.013 for control vs. 0.082 ± 0.016 for Gd^3+^, Figure 5A). However, much in line with the P2Y2 results, the addition of Pyr10 induced a significant decrease of calcium influx, around 66%, (Δ340/380 = 0.246 ± 0.038 for control vs. 0.083 ± 0.015 for Pyr10), as shown in Figure 5B. The major contribution of TRPC3 to PAR2 signaling was also demonstrated by shRNA-mediated knockdown experiments which showed a decrease in calcium influx similar to that of pharmacological inhibition with Pyr10 (Δ340/380 = 0.441 ± 0.025 for control vs. 0.149 ± 0.023 for TRPC3 shRNA, Figure 5C). This was further confirmed by the overexpression of heterologous TRPC3 that increased calcium influx more than twofold (Δ340/380 = 0.197 ± 0.012 for control vs. 0.463 ± 0.021 for TRPC3 OE, Figure 5D).

### PAR2- and P2Y2-mediated sensitization of nociceptors is TRPC3-dependent

**Figure 6:**
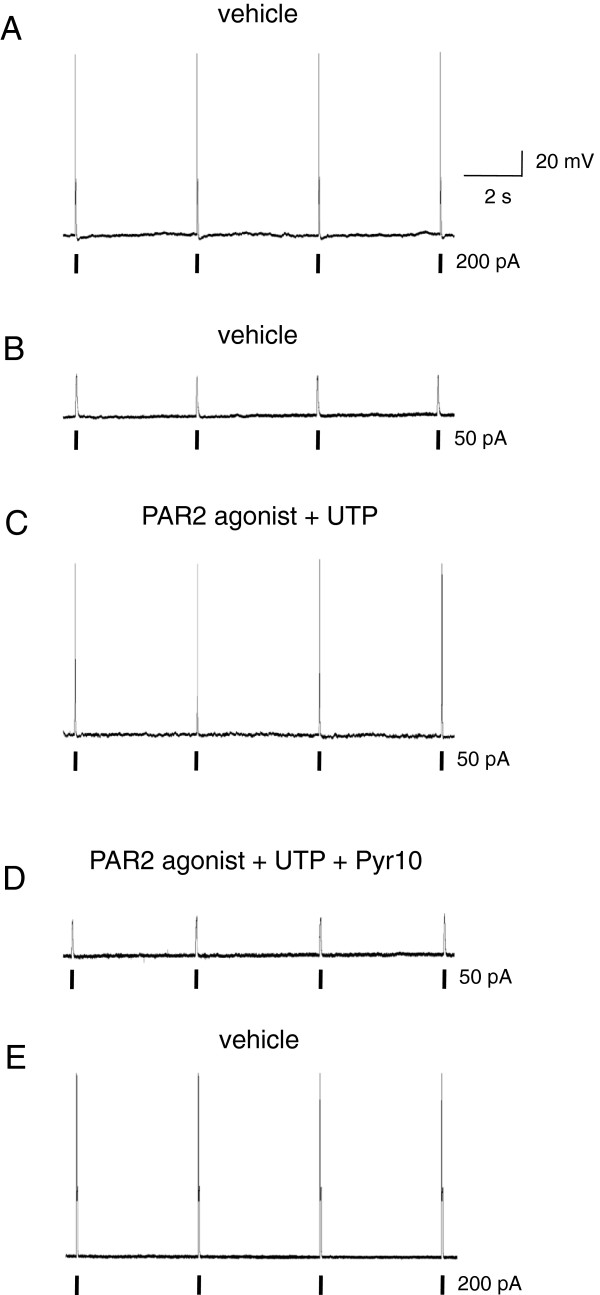
**TRPC3 is involved in peripheral sensitization induced by PAR2 and P2Y2 receptors.** Intrinsic excitability of a representative DRG nociceptor recorded sequentially under current-clamp conditions (RMP = -67 mV). **(A)** Firing of action potentials was consistently triggered by current pulses (200 pA; 50 ms duration) injected at fixed time intervals (n = 25). No firing was detected in conditions of subthreshold current injection (50 pA; 50 ms duration) **(B)** unless the cells were first sensitized by application of both PAR2 agonist AC55541 (100 μM) and P2Y2 agonist UTP (100 μM) (n = 14/25) **(C)**. **(D)** Treatment with the specific TRPC3 antagonist Pyr10 (10 μM) suppressed this sensitization effect in most neurons (n = 6/9). **(E)** At the end of each recording, cells were tested for viability and displayed normal firing activity after Pyr10 washout (n = 6).

We tested the contribution of TRPC3 to peripheral sensitization by sensitizing DRG neurons with the PAR2 agonist AC55541 and the P2Y2 agonist UTP. Patched cultured nociceptors showed a membrane resting potential between -58 mV and -70 mV. In current-clamp configuration, they were able to consistently fire action potentials during a current injection protocol (200 pA, 50 ms duration, 4 s intervals, Figure 6A). While current injection of 50 pA did not evoke action potentials in control conditions (Figure 6B), stimulation with both AC55541 (100 μM) and UTP (100 μM) sensitized 56% (14/25) of neurons to these subthreshold depolarizations (Figure 6C). Application of the selective TRPC3 antagonist Pyr10 (10 μM) suppressed 50 pA current-induced action potentials in 66.7% (6/9) of sensitized neurons (Figure 6D) in a reversible manner and without altering intrinsic excitability (Figure 6E). The application of Pyr10 alone did not affect resting membrane potential or firing properties (data not shown).

Taken together, these results strengthen the notion that TRPC3 channels are recruited in several pro-inflammatory metabotropic pathways, at least those triggered by the nucleotides ATP/UTP and trypsin-like proteases which are components of the “inflammatory soup”. TRPC3 channels also play a significant role in store- as well as receptor-operated calcium-dependent mechanisms in rat DRG neurons. The reported rise in basal intracellular calcium levels during inflammatory conditions is thought to mediate an increase in PKC activity, which has been shown to play a major role in neuronal sensitization [30,31,43]. TRPC3 contributes significantly to this process as shown by our *in vitro* sensitization data. Nevertheless, we cannot exclude a role of TRPC1 in the TRPC3-dependent ROCE and SOCE described here. Due to the documented heteromerization of store-linked TRPC1 with TRPC3 [17,34,35], it is likely that TRPC1 + 3 heteromers contribute to SOCE in DRG neurons as well. TRPC3 being a non-selective cation channel, its abnormal activation due to an upregulation of pro-inflammatory GPCRs such as P2Y2 and PAR2 could also mediate neuronal hyperexcitability through sustained depolarization. Although the conditional knockout of both TRPC3 and TRPC6 in Nav1.8+ DRG neurons did not induce behavioral deficits in acute pain responses [44], the mechanisms by which TRPC3 mediates calcium-dependent sensitization of nociceptors in inflammatory conditions will remain to be deciphered.

## Conclusions

Our data provide evidence that the DAG-gated and PLC-linked TRPC3 channel is involved in Orai-independent SOCE in adult rat primary nociceptors in DRG, where it is the major TRPC subunit expressed along with TRPC1. We show that in these neurons TRPC3 is also functionally coupled to several inflammatory transductions triggering calcium-dependent pathways and peripheral sensitization, including UTP/P2Y2 and proteases/PAR2 signaling complexes. We propose that this unique dual contribution to SOCE and ROCE defines the calcium-permeable TRPC3 channel as a key regulator of calcium homeostasis in DRG neurons in normal or pathological pain conditions.

## Methods

### Tissue extraction for RNA isolation

All experimental procedures were approved by the McGill University Animal Care Committee and were in compliance with the guidelines of the Canadian Council on Animal Care. DRGs were collected from adult Sprague-Dawley rats (4-8 weeks, Charles River) and suspended in Dulbecco’s modified Eagles medium containing 10% heat inactivated fetal bovine serum (Invitrogen), 1% penicillin and streptomycin, and 1% L-glutamine. Following extraction, DRG neurons were dissociated with fire-polished glass pipettes and sieved on 40 μm filters, thus limiting primary cultures to small and medium diameter neurons. Dissociated neurons were plated on poly-D-lysine and laminin coated 60 mm tissue culture dishes, and left overnight at 37°C and 5% CO_2_ to recover. The following day, cells were collected with 1% trypsin-EDTA and centrifuged. The pellet was used to extract total RNA using Qiagen’s RNeasy Mini Kit as described by the manufacturer. To eliminate possible genomic DNA contamination, total RNA samples were treated with RNase-Free DNase (Qiagen) in accordance with the manufacturer’s protocol.

### cDNA synthesis and RT-PCR

Total RNA DRG extracts were used as templates to synthesize single-strand cDNAs. Random hexamers (Invitrogen) and RNaseOUT Recombinant Ribonuclease Inhibitor (Invitrogen) were used with the Omniscript RT Kit (Qiagen) to reverse-transcribe the cDNA templates according to the manufacturer’s recommendations. cDNAs were used in subsequent PCR reactions to determine the expression of TRPC gene family members in adult rat DRGs.

### *In situ* hybridization

Rat tissue was obtained from adult male Sprague-Dawley rats (n = 3). TRPC1 and TRPC3 mRNAs were detected by radioactive *in situ* hybridization (ISH), as previously described [45]. Anti-sense ^35^S-radiolabeled riboprobes were directed to the 1616-2177, 2246-2706, 1269-1797, 1561-1976, 2129-2512, 1891-2207 and 1797-2560 sequences of the rat TRPC 1, 2, 3, 4, 5, 6, 7 mRNA, respectively. These riboprobes were designed to detect selectively each TRPC subunit transcript and sense probes were used as negative controls.

### Primary cultures for calcium imaging and electrophysiology

DRG extraction (n = 30-40) was carried out on 1-2 month old Sprague-Dawley rats. Following extraction, DRG neurons were dissociated with fire-polished glass pipettes, filtered with 40 μm filters, and suspended in Dulbecco’s modified Eagles medium (DMEM) containing 10% heat inactivated fetal bovine serum (Invitrogen), 1% penicillin and streptomycin, and 1% L-glutamine. Nontransfected homogenized DRG neurons were plated on 35 mm glass bottom dishes coated with poly-D-lysine (Sigma-Aldrich) and laminin (BD Bioscience) at a density of 50-100,000 cells/dish, and incubated with 2 ml of complete DMEM media, at 37°C in 5% CO_2_ until recording and imaging.

For patch-clamp recording, DRG ganglia were mechanically triturated using fire-polished Pasteur pipettes as well, but after each trituration, partially dissociated cells were briefly centrifuged (1000 rpm), and the supernatant was collected. Dissociated cells were plated on 35-mm culture dishes (Starstedt; 2 ml/dish) coated previously with laminin and poly-D-lysine. Cells were incubated for 24 h to 48 h at 37°C in 5% CO_2_ before electrophysiological recording.

### Overexpression and shRNA-mediated knockdown

Adult rat DRGs (n = 35-40) were transiently transfected using Amaxa Rat Neuron Nucleofector Kit (Lonza) and Nucleofector I (Amaxa), in accordance with the manufacturer’s guidelines. Nucleofected neurons were plated on 35 mm glass bottom dishes coated with poly-D-lysine and laminin for subsequent calcium imaging. Cultured neurons were maintained in Neurobasal-A medium (Invitrogen) supplemented with B-27 (Invitrogen), 1% penicillin and streptenomycin, and 1% L-glutamine, 17.5 μg/ml uridine (Sigma-Aldrich) and 7.5 μg/ml of 5-fluoro-2’-deoxyuridine (Sigma-Aldrich). Media was replaced every 48-72 hours and cultures were incubated at 37°C in 5% CO_2_.

Heterologous expression of TRPC3 was induced with co-nucleofection of 6 μg of mouse TRPC3 in pcDNA3 along with 2 μg of GFP plasmid. Recording experiments were carried out on transfected GFP+ DRG neurons 48-72 hours post-transfection. TRPC3-specific shRNA construct (Origene) was used to interfere with the translation of the endogenous TRPC3 subunits as previously described [46]. Knockdown was performed by transient transfection of 6 μg GFP-tagged TRPC3 shRNA using Nucleofector I as described above. Cultures were incubated at 37°C in 5% CO_2_ for a period of 4-6 days for maximal knockdown before recording.

### Calcium imaging

DRG neurons were plated on glass bottom dishes coated with poly-D-lysine- and laminin-coated dishes at a density of 50-100,000 cells/dish. Prior to recording, the cells were loaded with 5 μM Fura-2 AM (Molecular Probes) + 0.1% BSA for 40 min and then washed for 30 min with the extracellular solution (containing in mM: 152 NaCl, 5 KCl, 2 CaCl_2_, 1 MgCl_2_, 10 HEPES and 10 glucose, pH 7.4) at 37°C in 5% CO_2_. During recording, the cells were constantly perfused with the extracellular solution and stimulated with the appropriate agonist. Cells were selected using an inverted TE2000-U microscope (Nikon) equipped with 40X oil-immersion objective [CFI super(S) fluor, Nikon]. Fura-2 AM was excited at 340 nm and 380 nm every second and emission at 510 nm was detected by a high-resolution cooled CCD camera (Cool Snap-HQ, Roper Scientific/Photometrics) interfaced to a Pentium III PC. The variation in intracellular calcium levels was determined by the ratio of fluorescence at 340 nm and 380 nm (340/380 ratio) calculated using the Metafluor 7.0 software (Molecular Devices). For each cell, agonist-induced increase in intracellular calcium (Δ340/380) was determined by subtracting the baseline ratio from the peak ratio of the response, divided by the baseline. All experiments were conducted at room temperature. The SOCE and TRPC blocker SKF96365 (Tocris) was used. The compound Pyr10, a selective TRPC3 antagonist, was provided by Dr. Groschner (Institute of Biophysics, Medical University of Graz, Austria) [47]. Store-operated calcium signaling was induced in cultured DRGs by applying the sarco/endoplasmic reticulum Ca^2+^ ATPase blocker thapsigargin (1 μM) for 7 minutes in calcium-free perfusion solution. Fluctuation of intracellular calcium levels was measured using single-cell microfluorescence. The P2Y2 agonist UTP (Tocris) and the small-molecule PAR2 agonist AC55541 (Tocris) were added to the calcium-free perfusion solution (100 μM for 7 min) to activate their respective Gq-coupled receptors in DRG neurons.

### Patch-clamp electrophysiology

Whole-cell patch-clamp recordings on DRG neurons were conducted 24 hr post plating at room temperature. The internal solution of the pipette, pH 7.2, contained (in mM): 130 K-gluconate, 1 MgCl_2_, 10 HEPES, 5 EGTA, 3 MgATP, and 0.4 GTP. The bath solution, pH 7.4, contained (in mM): 152 NaCl, 5 KCl, 2 CaCl_2_, 1 MgCl_2_, 10 HEPES, and 10 glucose. Patch pipettes had a tip resistance of 3–8 MΩ. Electrophysiological recording experiments were performed using an Axopatch 200B amplifier and digitized with a Digidata 1322A interface (Molecular Devices). Traces were acquired and analyzed using pClamp 8.2 software (Molecular Devices). Recordings were low-pass filtered at 2 kHz and 5 kHz in voltage- and current-clamp configurations, respectively. Under current-clamp, action potentials were electrically-induced (50-200 pA) with 50 ms depolarizing pulses at fixed time intervals.

### Data analysis

Student’s *t* tests were used for assessing statistical significance between two experimental conditions. Differences were considered significant at *P* < 0.05.

## Competing interests

The authors declare that they have no competing interests.

## Authors’ contributions

PS conceived the project and participated in its design and coordination. RG and DOD carried out the *in situ* hybridization experiments and emulsion stains. ARA designed the protocol of single-cell calcium imaging for ROCE and SOCE, as well as performing all patch clamp recordings. HA performed calcium imaging experiments and data analysis on DRG neurons. KG provided the compound Pyr10 and comments on the manuscript. HA, PS and ARA wrote the manuscript. All authors read and approved the final manuscript.
